# Albumin-bioinspired iridium oxide nanoplatform with high photothermal conversion efficiency for synergistic chemo-photothermal of osteosarcoma

**DOI:** 10.1080/10717544.2019.1662513

**Published:** 2019-09-16

**Authors:** Wenguang Gu, Tao Zhang, Junsheng Gao, Yi Wang, Dejian Li, Ziwen Zhao, Bo Jiang, Zhiwei Dong, Hui Liu

**Affiliations:** a Department of Orthopedics, The First Affiliated Hospital of Harbin Medical University, Harbin, China;; b Department of Orthopedics, Shanghai Pudong Hospital, Fudan University Pudong Medical Center, Shanghai, China;; c Department of Cardiology, The First Affiliated Hospital of Harbin Medical University, Harbin, China

**Keywords:** Iridium oxide, bovine serum albumin, photothermal, biomineralization, synergistic therapy

## Abstract

Protein-based nanocarriers with inherent biocompatibility have been widely served as building blocks to construct versatile therapeutic nanoplatforms. Herein, bovine serum albumin-iridium oxide nanoparticles (denoted BSA-IrO_2_ NPs) are successfully synthesized *via* one-step biomineralization approach. The BSA-IrO_2_ NPs exhibits uniform size (40 nm), superb biocompatibility and high drug loading capacity for doxorubicin (27.4 wt%). Under near-infrared (NIR) laser irradiation, the as-prepared BSA-IrO_2_ NPs exhibited high photothermal conversion ability (54.3%) and good photostability. The *in vitro* drug release experiments displayed pH and NIR laser -triggered DOX release profiles, which could enhance the therapeutic anticancer effect. By utilizing this DOX loaded nanoplatform, effective synergistic chemo-photothermal therapy against human osteosarcoma can be realized, which has been systematically verified both *in vitro* and *in vivo*. Notably, *in vivo* pharmacokinetics studies showed that BSA-IrO_2_@DOX had prolonged blood circulation time due to the BSA component can improve the stealthiness of the nanoparticles during the blood circulation. Meanwhile,* in vitro* and *in vivo* toxicity studies demonstrated that the BSA-IrO_2_ NPs can act as biocompatible agents for drug delivery and cancer therapy. Therefore, this work presents a biomineralized iridium-based NPs with remarkable features and be used as a very potential therapeutic nanoplatform for cancer treatment.

## Introduction

Cancer becomes one of the primary hazards to human health worldwide. With the developments in our understanding of cancer biology, tremendous effective methods have been extensively explored for cancer therapy, including chemotherapy, radiotherapy, and surgery (Garg & Buchholz 2015; Landoni et al., 2017; Bray et al., 2018). As a classic and effective strategy, chemotherapy is widely applied in the clinic. However, it usually limited by the low stability and hydrophobicity of many chemotherapeutics (Shi et al., 2017), as well as nonspecific adsorption and biodistribution, thus leading to significant systemic toxicity. In recent years, nanocarriers have attracted much attention in drug delivery to enhance the bioavailability of drugs through sustained drug release at the targeted tumor tissue and avert non selectivity uptake by the reticuloendothelial system (RES) (Lu et al., 2016; Pan et al., 2017; Bose et al., 2018). Also, long blood circulation time of therapeutic nanoplatforms is the prerequisite for their therapeutic potential (Wang et al., 2019). As far as nanomedicine is concerned, nanoparticles with appropriate sizes of 50–100 nm are propitious to prolong blood circulation time, as well as efficient tumor accumulation by the enhanced permeability and retention (EPR) effect (Tang et al., 2013; Chen et al., 2016a). Therefore, it is of great significance to fabricate an ideal therapeutic nanoplatform with prolonged blood circulation time and high drug loading efficiency.

On the other hand, based on the cooperative improvement interactions between two or more therapeutic modalities, recent advances in clinical research have gradually turned from a focus on monotherapy to combination therapy for improving therapeutic effects (Kim et al., 2016; Fan et al., 2017). Among of them, the combination of chemotherapy and photothermal therapy (PTT) has been well documented (Huang et al., 2017; Peng et al., 2018; Wu et al., 2018b). As an emerging efficient modality, PTT-enhanced chemotherapy holds great potential to reduce the adverse effects and enhance the treatment efficacy (Lu et al., 2017). The combination of chemotherapy and PTT offers many advantages for enhancing the therapeutic outcomes. It has been proved that some anticancer drugs may exhibit increased cytotoxicity upon high temperature induced by the photothermal agents (Hauck et al., 2008). Meanwhile, hyperthermia can dramatically improve tumor cell uptake of nanoparticles, thus significantly elevating drug cytotoxicity (Li et al., 2015; Pacardo et al., 2015). Thus, many nanosystems have been designed for synergistic chemo/PTT treatment due to the excellent anticancer efficacy.

To fabricate a smart chemo/PTT nanoplatform, an ideal nanocarrier is a primary step to achieve the combination of various therapeutic elements into one system. In the past years, many nanomaterials, including mesoporous silica (Wu et al., 2018a), mesoporous carbon (Augustine et al., 2017), 2 D nanomaterials (Yang et al., 2018), graphene (Chen et al., 2016b), and transition-metal dichalcogenide/oxide nanoparticles (Chen et al., 2015) have been utilized as carriers to build the nanoplatforms for synergistic chemo/PTT treatment. Among various types of functional nanosystems explored in this application, protein-based nanocarriers with inherent biocompatibility have been widely served as building blocks to construct versatile therapeutic nanoplatforms (Gao et al., 2017; Yang et al., 2017; Li et al., 2018). Albumin, the most abundant type of plasma protein has been extensively reported as a versatile drug delivery carrier due to its good biocompatibility (Chen & Liu 2016). Thanks to the abundant charged amino acids, together with unique structure with both hydrophilic and hydrophobic domains, drug molecules can be connected with albumin *via* electrostatic/hydrophobic interaction or covalent conjugation (Kratz 2008). Abraxane, a FDA approved anti-cancer drug, is fabricated by loading paclitaxel (PTX) to HSA via hydrophobic interactions, which has been approved for treatment of many different types of cancers (Green et al., 2006). Furthermore, docetaxel and rapamycin loaded in albumin to achieve other albumin-based drugs for cancer treatment, which are advancing into clinical trials (Cirstea et al., 2010; Jiang et al., 2015). Besides binding with drug molecules, albumin could be used to assist in the preparation of various ultrasmall size inorganic metal sulfide (e.g. CuS, Ag_2_S, Bi_2_S_3_, and CdS) NPs by using albumin as both a sulfur donator and a template (Li et al., 2016; Gao et al., 2017; Sheng et al., 2018). Furthermore, albumin is able to sequester inorganic ions to form protein coated metal oxide nanoclusters in alkaline conditions via a mild biomineralization strategy. For instance, Liu and coworkers successfully designed a multifunctional dual-responsive HSA-coated MnO_2_ nanoplatform through albumin-based biomineralization of Mn^2+^ to overcome tumor hypoxia-associated resistance of PDT, thus can be used for effective combination therapy (Chen et al., 2016a). Also, Wang et al. developed an albumin-based nanotherapeutic agent by cypate-grafted gadolinium oxide nanocrystals through a biomineralization approach for multimode imaging-guided photothermal therapy (Wang et al., 2015). Thus, the construction of all-in-one nanoplatform integrating metal oxide nanoclusters and another therapeutic agent via a biomineralization process strategy is favorable for enhancing anticancer efficiency.

**Figure 1.**
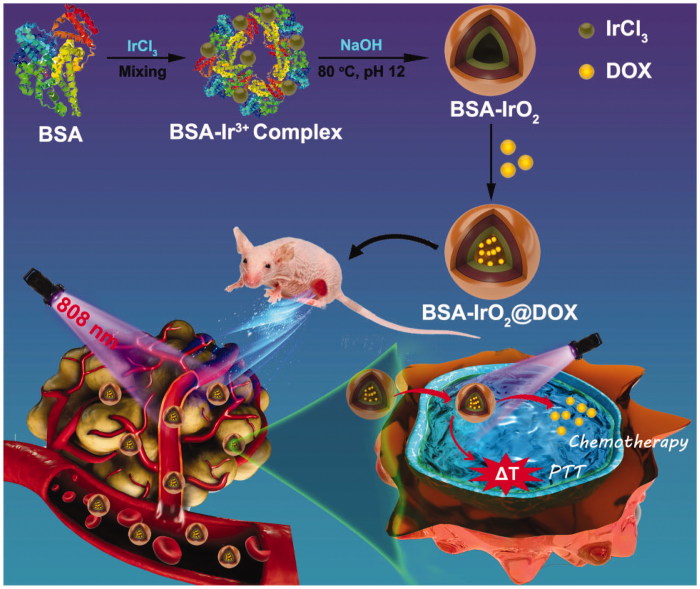
Schematic illustration of the preparation of BSA-IrO_2_@DOX and served as a versatile nanoplatform for mild hyperthermia-induced DOX release and synergistic chemo-photothermal therapy.

Herein, we design a smart therapeutic platform through bovine serum albumin (BSA)-based biomineralization strategy by incubating iridium ion (Ir^3+^) with BSA in alkaline conditions. In this process, Ir^3+^ would be anchored to BSA to form albumin-Ir complexes through the affinity of the active groups (amino and carboxy groups) of BSA toward metal ions. Subsequently, the pH value of system was adjusted to ∼12 with NaOH and Ir^3+^ is oxidized into IrO_2_. The resultant BSA-iridium oxide nanoparticles (BSA-IrO_2_ NPs) were then used as a drug nanocarrier to load chemotherapeutic agent doxorubicin (DOX) for chemotherapy. Meanwhile, the BSA-IrO_2_ NPs can be also served as photothermal agents for cancer PTT due to their superior photothermal conversion efficiency of BSA-IrO_2_ NPs (54.3%). Based on this, the as-prepared BSA-IrO_2_@DOX NPs were then used as a therapeutic nanoplatform for cancer chemo-photothermal synergistic therapy (Figure 1). The combination of chemotherapy and PTT under NIR laser irradiation resulted in significant tumor growth suppression, which has been systematically demonstrated both *in vitro* and *in vivo*. Therefore, this work presents a biomineralized iridium-based NPs as remarkable therapeutic nanoplatform with significant clinical values.

## Materials and methods

### Materials

Bovine serum albumin (BSA) and sodium hydroxide (NaOH) were purchased from Sinopharm Chemical Reagent Co., Ltd (Shanghai, China). Iridium trichloride (IrCl_3_) was acquired from Aladdin Reagent Co. (Shanghai, China). Doxorubicin hydrochloride (DOX, purity 98%), phosphate buffered saline (PBS), fetal bovine serum (FBS), calcein acetoxymethyl ester (Calcein AM) and propidium iodide (PI) were obtained from Sigma-Aldrich (St. Louis, MO, USA). Cell counting lit-8 (CCK-8) was ordered from Dojindo Laboratory (Dojindo Co., Ltd. Japan). Dulbecco’s modified Eagle medium (DMEM), penicillin, and streptomycin were obtained from Gibco (Invitrogen, Carlsbad, CA). The Human osteosarcoma cancer cell line (Saos-2 cells) and A549 cells were obtained from the Institute of Basic Medical Sciences Chinese Academy of Medical Sciences (Shanghai, China). Milli-Q water was prepared using a Milli-Q system (Bedford, MA, America). All chemicals were used as received without further purification.

### Preparation of BSA-IrO_2_ NPs

BSA-IrO_2_ NPs were prepared according to an one pot biomineralization approach (Zhen et al., 2018). Briefly, solution A: BSA (200 mg) was first dissolved in 10.0 mL of deionized water. Solution B: IrCl_3_ (60 mg) was dissolved in 4 mL of deionized water. Then, 10.0 mL of solution A and 2.0 mL of solution B were mixed adequately and stirred for 30 min. Subsequently, a certain volume of NaOH solution (2.0 M) was introduced to adjust the pH of the mixture to ∼12, and the mixture was stirred at 80 °C. After 12 h reaction, the solution was dialyzed (membrane cutoff Mw: 100 KD) against deionized water for 48 h to remove excess precursors. Finally, the mixture was freeze-dried, harvested and re-dispersed in 10 mL of water for further use.

### Preparation of BSA-IrO_2_ therapeutic delivery nanoplatform (BSA-IrO_2_@DOX)

BSA-IrO_2_@DOX NPs were fabricated by the absorption method. 5 mg of BSA-IrO_2_ NPs were dispersed into 10 mL of phosphate buffer saline (PBS) and then different concentrations of DOX solution (0.2–1.0 mg/mL) were mixed. After stirring for 24 h under dark condition at room temperature, the resulting BSA-IrO_2_@DOX NPs were collected by centrifugation and the product was washed with PBS repeatedly to remove the excess DOX. Meanwhile, the supernatant was collected and measured by UV-Vis spectroscopy at 490 nm. The DOX loading capacity (LC) was calculated as: (the initial feeding amount of DOX-the DOX content in the supernatant)/the amount of BSA-IrO_2_.

### Characterization

The morphology of NPs was observed by JEOL-2100 transmission electron microscopy (TEM, JEOL, Japan). UV-1800 Spectrophotometer was used to record UV-vis absorption spectra with a 1 cm cuvette (Shimadzu, Japan). An IR Prestige-21 spectrometer was used to record the Fourier transform infrared (FTIR) spectrum (Shimadzu, Japan). X-ray photoelectron spectras (XPS) were measured with EscaLab 250Xi electron spectrometer from VG Scientific using 300 W Al Kα radiations (Thermo Fisher Scientific, USA). The hydrodynamic diameters and Zeta potential were conducted on Malvern Zetasizer Nanoseries (Nano ZS90, Malvern, UK). The content of Ir was detected by inductively coupled plasma-atomic emission spectroscopy (ICP-AES, Agilent Technologies). Thermal images were also captured with the TI100 infrared thermal imaging camera (FLK-TI100 9HZ, FLUKE).

### Photothermal properties of the BSA-IrO_2_ NPs

To examine the photothermal conversion performance of BSA-IrO_2_ NPs, 0.2 mL of BSA-IrO_2_ NPs PBS aqueous solutions with different concentrations (Ir: 0.5–6 mM) were introduced into a centrifugation tube and fixed on a retort stand. All samples were irradiated with an 808 nm NIR laser (SFOLT Co., Ltd, Shanghai, China) at power density of 1.0 W cm^−2^ for 5 min. Also, the BSA-IrO_2_ NPs dispersion at Ir concentration of 3 mM was exposed with a different power density (0.3, 0.5, 0.8, 1.0 and 1.2 W cm^−2^) over a period of 5 min. The temperature changes of dispersion were recorded using a digital thermometer (Shenzhen Everbest Machinery Industry, Shenzhen, China) and infrared thermal imaging camera. Meanwhile, the thermal stability of BSA-IrO_2_ was evaluated by irradiating for 5 min each time with five on-off cycles. Also, the photothermal conversion efficiency (η) of BSA-IrO_2_ NPs was calculated by the previous methods (Ji et al., 2018).

### *In vitro* drug release

To investigate the pH and NIR laser promoted DOX drug release, the dispersion of BSA-IrO_2_@DOX (2 mg mL^−1^, 3 mL) in PBS buffers was divided into four groups: (a) pH 5.0 in dark, (b) pH 7.4 in dark, (c) pH 5.0 and (d) pH 7.4 with irradiation (808 nm, 1.0 W cm^−2^, 5 min) at different time intervals (5, 40 and 90 min). At the indicated time interval, 0.2 mL of NPs suspension was taken out, centrifuged at 10000 rpm for 10 min and equal volume of PBS was added to the system again. The cumulative amount of released DOX from the BSA-IrO_2_@DOX was determined according to the UV-Vis absorption of DOX at 490 nm in the supernatant. All the release experiments were repeated three times.

### Cell culture and biocompatibility of BSA-IrO_2_ NPs

Saos-2 and A549 cells were incubated in DMEM medium supplemented with 10% FBS and 1% antibiotics (penicillin-streptomycin), and cultured in a humidified atmosphere with 5% CO_2_ at 37 °C. To evaluate the cytotoxicity of BSA-IrO_2_ NPs, Saos-2 and A549 cells (5000 cells/well) seeded in 96-well plates and incubated for 24 h. Subsequently, the old culture medium was replaced with fresh culture medium (200 μL) containing different concentrations of BSA-IrO_2_ NPs (Ir: 0, 0.5, 1.0, 1.5, 3.0, 6.0, and 10 mM) and incubation for another 24 h. The cells were washed with PBS for three times and the cell viability was determined by using the CCK-8 proliferation assay according to the manufacture’s protocol.

### *In vitro* cellular uptake of BSA-IrO_2_@DOX NPs

The cellular uptake of BSA-IrO_2_@DOX was evaluated by confocal laser scanning microscopy (CLSM), flow cytometry and ICP-AES analysis. For CLSM, Saos-2 cells were seeded into confocal culture dish with a density of 5 × 10^5^ cells/mL^−1^ and incubated for 24 h, followed by the addition of BSA-IrO_2_@DOX or free DOX (DOX: 5 μg mL^−1^) with or without 5 min laser irradiation (after 2 h incubation) and an incubation for another 6 h. After that, the cells were washed with PBS and stained with DAPI (2 μg mL^−1^) for 20 min. The fluorescent images were obtained by using a CLSM (Leica TCS SP5, Germany). For the flow cytometry analysis, Saos-2 cells were seeded in glass-bottom culture dishes (1 × 10^5^ cells per dish) for 24 h, followed by the addition of free DOX and BSA-IrO_2_@DOX at a DOX concentration of 5 µg mL^−1^ with or without 5 min laser irradiation (after 2 h incubation) and an incubation for another 6 h. Afterward, the cells in all groups were digested with trypsin, followed by centrifugation. After washing by PBS and resuspending in PBS, the intracellular fluorescence of DOX was studied by using a FACScan flow cytometry (Becton Dickinson, CA, USA). Furthermore, half of the cells were washed, trypsinized, and digested by 1 mL of aqua regia overnight and diluted with 1 mL of water. Then, the intracellular Ir content in every cell samples was determined by ICP-AES assay.

### *In vitro* chemo-PTT synergistic effect of saos-2 cells

To evaluate the combined PTT and chemotherapy of BSA-IrO_2_@DOX NPs, Saos-2 cells were seeded in 96-well plates (1 × 10^4^ cells/well) and incubated for 24 h. The cells were incubated with different concentrations of DOX, BSA-IrO_2_ and BSA-IrO_2_@DOX. After that, part of cells treated with BSA-IrO_2_ and BSA-IrO_2_@DOX were irradiated with an 808 nm laser (1.0 W cm^−2^, 5 min) after 6 h incubation. The cells were then incubated for totally 24 h and the cell viability was measured by using the CCK-8 proliferation assay.

The in vitro antitumor efficiency of BSA-IrO_2_@DOX NPs was evaluated by cell live/dead assays. Saos-2 cells were seeded onto 6-well plates (5 × 10^5^ cells per well) and treated as mentioned previously. After that, the cells after different treatments were washed with PBS, stained by calcein-AM (5 μg mL^−1^) and PI (10 μg mL^−1^) and incubated for 30 min at 37 °C. Then, the cells were washed with PBS for three times and imaged by an inverted fluorescence microscope (Nikon ECLIPSE Ts2R).

### Animal tumor model

Female Balb/c nude mice (15–20 g) and female Kunming mice (200-220 g) were provided by the Beijing Vital River Laboratory Animal Technology Co., Ltd. All animal experiments were approved by the institutional ethical committee of the Harbin Medical University. The tumor models were set up by subcutaneous injection of Saos-2 cells (5 × 10^6^) on the right flank of each mouse. Mice were used for the following in vivo experiments when the tumor volume grew to ∼100 mm^3^.

### *In vivo* therapeutic efficacy

The Saos-2 tumor-bearing mice were randomly divided into six treatment groups (*n* = 5) as follows: (1) PBS, (2) BSA-IrO_2_ NPs, (3) free DOX (5 mg kg^−1^), (4) BSA-IrO_2_@DOX NPs (5 mg kg^−1^ of DOX), (5) BSA-IrO_2_ NPs + laser and (6) BSA-IrO_2_@DOX NPs (5 mg kg^−1^ of DOX) + laser. Mice in laser irradiation group were exposed to 808 nm laser of 1.0 W/cm^2^ for 5 min after 12 h intravenous injection of NPs. Meanwhile, the temperature changes of the tumor sites were monitored using an infrared thermal camera during the treatment. The body weight and tumor size were measured every 3 days. The tumor volume was calculated by following equation: Volume = (length × width^2^)/2.

### Histological examination

After 14 days of treatment, mice of all groups were sacrificed, and their major organs (liver, heart, lung, spleen, and kidney) and tumor were removed. The tumors were harvested, fixed in a 10% formalin solution, and embedded in paraffin for hematoxylin and eosin (H&E) and deoxynucleotidyltransferase-mediated dUTP nick end labeling (TUNEL) staining. To investigate the *in vivo* toxicity, the major organs, including hearts, livers, spleens, lungs, kidneys from different treatments were further stained with H&E for histological analysis.

### *In vivo* blood circulation and biodistribution

For pharmacokinetic analysis, female Kunming mice were intravenously injected with BSA-IrO_2_@DOX NPs (20 mg kg^−1^) in PBS (n = 4). At certain designated time intervals (10 min, 30 min, 1, 2, 3, 4, 6, 8, 12, 24 h), ∼20 μL of blood was withdrawn from the treated mice and put into physiological saline (1 mL) containing heparin sodium (50 unit mL^−1^). The samples were then digested with aqua regia (HNO_3_: HCl = 1:3), and the amount of Ir in the blood was measured by ICP-AES. The blood terminal half-life of the BSA-IrO_2_@DOX NPs was calculated by using a two-compartment model. For the in vivo biodistribution study, the Saos-2 tumor-bearing mice (*n* = 3) were intravenously injected with BSA-IrO_2_@DOX (20 mg kg^−1^) in PBS. At varied time intervals (4, 12, and 24 h), the tumor and main organs (heart, liver, spleen, lung, and kidney) of the mice were collected and homogenized, followed by ICP-AES measurement for measuring Ir concentration. The distribution of NPs in tumor and different organs were calculated as the percentage of injected dose per gram of tissue.

### Statistical analysis

Experimental results were expressed as the mean ± standard deviation, and an analysis of variance (ANOVA) test was performed for statistical analysis. *p* < .05 was considered statistically significant (SPSS, Chicago, IL).

## Results and discussion

### Preparation and characterization of BSA-IrO_2_@DOX NPs

**Figure 2.**
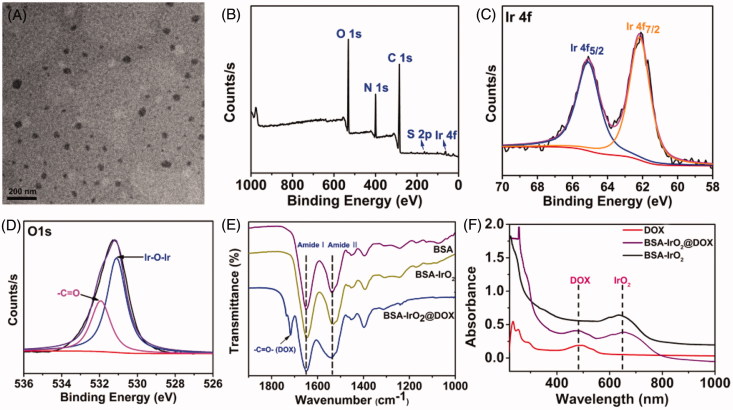
Preparation and characterization of BSA-IrO_2_@DOX NPs. (A) TEM images of BSA-IrO_2_ NPs. (B) The XPS survey spectrum of BSA-IrO_2_ NPs and the selective XPS survey spectrum corresponding to (C) Ir 4f spectra and (D) O element of BSA-IrO_2_ NPs. (E) FT-IR spectra of BSA, BSA-IrO_2_ and BSA-IrO_2_@DOX NPs. (F) UV-Vis spectra of DOX, BSA-IrO_2_ and BSA-IrO_2_@DOX NPs.

In this work, multifunctional biomineralized iridium-based (BSA-IrO_2_@DOX) NPs as therapeutic nanoplatform were fabricated for synergistic chemo-photothermal of tumor, which is illustrated in Figure 1. BSA-IrO_2_ NPs was prepared by incubating iridium ion (Ir^3+^) with BSA in alkaline conditions through biomineralization process, where BSA acted as the stabilizer and template. In this process, BSA in water was mixed with IrCl_3_ through electrostatic interactions between Ir^3+^ ions and the active groups (amino and carboxy groups) in BSA due to the great affinity of these groups. Subsequently, the pH value of system was adjusted to ∼12 with NaOH to cause expansive unfolded conformation and Ir^3+^ is oxidized into IrO_2_. Upon the introduction of NaOH, the mixed solution rapidly became purple-blue color (Figure S1), which may be attributed to the conformational transformation of protein (Wang et al., 2015; Yang et al., 2016). TEM images (Figure 2(A)) demonstrated the sphere-like structure of the obtained BSA-IrO_2_ NPs with an average diameter of 41 nm. The X-ray photoelectron spectroscopy (XPS) of BSA-IrO_2_ NPs demonstrated the existence of Ir, C, N, S, and O (Figure 2(B)). Also, the characteristic peaks at 62.2 and 65.1 eV were observed in the high-resolution XPS spectra of Ir 4f (Figure 2(C)), which could be assigned to the Ir 4f _7/2_ and Ir 4f _5/2_ (Zhen et al., 2018). There were two peaks at 532.0 and 531.2 eV in the XPS spectra of O1s (Figure 2(D)), which are attributed to the oxygen in –COOH, –C=O of BSA and Ir–O–Ir bonding, respectively (Yang et al., 2016). These results confirmed the formation of BSA-IrO_2_. Figure 2(E) show the FTIR spectras of BSA and BSA-IrO_2_ NPs, which show nearly identical amide I and amide II bands at 1650 cm^−1^ and 1550 cm^−1^, suggesting the successful coating of BSA. Subsequently, the fabricated NPs can be dispersed well in water with a hydrodynamic size of 62 ± 3.2 nm and zeta-potential of −30.4 mV (Figure S2), which are propitious to prolong blood circulation time in the biomedical application.

Since BSA have been proved as drug carriers to interact with various drug molecules (Chen & Liu 2016), the potential of BSA-IrO_2_ NPs as drug carriers was further evaluated by using DOX as the model drug. BSA-IrO_2_ NPs were mixed with the different concentrations of DOX (0.2–1.0 mg mL^−1^) for 24 h at room temperature. The successful loading of DOX i was confirmed by UV-Vis spectroscopy and FT-IR spectra. From the UV-Vis spectrum of BSA-IrO_2_@DOX (Figure 2(F)), the characteristic absorption peak of DOX at 490 nm was observed, while no absorption peak was appeared at this wavelength. Also, the FT-IR spectrum of BSA-IrO_2_@DOX NPs shows a new band at 1716 cm^−1^, which was assigned to C=O carbonyl stretching of DOX (Figure 2(E)). These results demonstrated that the DOX was loaded into the BSA-IrO_2_. The zeta potential of BSA-IrO_2_@DOX changed to −17.6 mV after loading of DOX (Figure S2), which is due to the positively charged of DOX. Simultaneously, the hydrodynamic sizes of BSA-IrO_2_@DOX were increased to about 84.5 ± 2.1 nm, which is slightly increased compared to BSA-IrO_2_ (Figure S2). As shown in Figure S3, the saturation of DOX loading was reached to be 27.40% by weight. The BSA-IrO_2_@DOX NPs could be also well dispersed in PBS and cell culture medium, and the hydrodynamic size was almost unchanged after standing for 72 h, confirming the excellent stability of BSA-IrO_2_@DOX NPs (Figure S4).

### Photothermal properties of the BSA-IrO_2_ NPs

**Figure 3.**
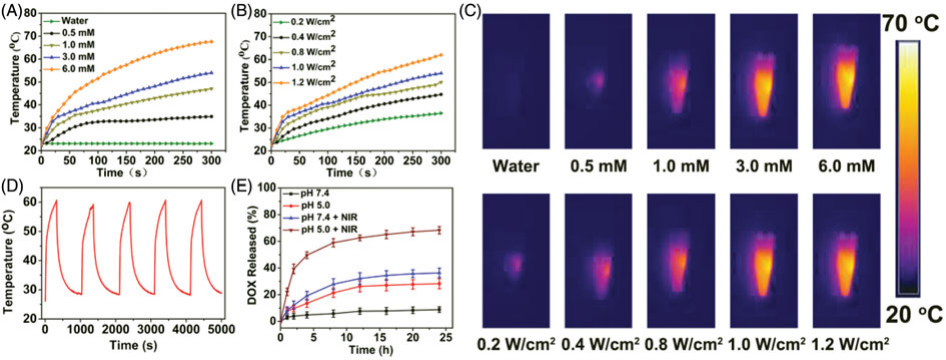
Photothermal property of the BSA-IrO_2_ NPs and DOX release from BSA-IrO_2_@DOX at different conditions. (A) Photothermal effect of BSA-IrO_2_ as a function of NIR laser irradiation time (808 nm, 1.0 W cm^−2^) with different concentrations of Ir. (B) Photothermal effect of BSA-IrO_2_ as a function of NIR laser irradiation time (Ir: 3.0 mM) with different power densities. (C) Infrared thermal images of BSA-IrO_2_ aqueous solutions irradiated with an 808 nm laser at varied concentrations or different power densities. (D) Temperature records of BSA-IrO_2_ NPs aqueous solution (6 mM) after five cycles of laser on/off at 1.0 W cm^−2^. (E) Release profiles of DOX at different pH with or without 808 nm NIR laser (1.0 W cm^−2^).

Due to BSA-IrO_2_ NPs displays high absorbance in the near-infrared (NIR) region with a maximal peak at 634 nm (Figure 2(F)), the photothermal properties of the BSA-IrO_2_ NPs were evaluated. The photothermal effect was dependent on the laser power density and the concentration of BSA-IrO_2_ (Figure 3(A,B)). The temperature of the dispersion of BSA-IrO_2_ (6 mM of Ir) could be increased to 69.8 °C after 5 min laser irradiation (1.2 Wcm^−2^). Meanwhile, the strong photothermal effect also provided excellent signal contrast for infrared thermal imaging (Figure 3(C)). Notably, there is no obvious change of the temperature response of the BSA-IrO_2_ dispersion after five on-off cycles of laser irradiation, indicated the excellent photothermal stability of BSA-IrO_2_ NPs (Figure 3(D)). The photothermal conversion effciency (η) of BSA-IrO_2_ NPs was also determined by the previously reported methods (Ji et al., 2018). The photothermal conversion efficiencies are calculated to be 54.3% for BSA-IrO_2_ NPs, which is higher than that of the majority of good photothermal agents (Cheng et al., 2014; Lei et al., 2017; Liu et al., 2017; Cao et al., 2018). These results confirmed that BSA-IrO_2_ NPs possessed the superior ability of NIR photothermal transduction.

### *In vitro* drug release

The feasibility of the pH and NIR laser-triggered DOX release from BSA-IrO_2_@DOX NPs was investigated at different pH (7.4 and 5.0) in the presence or absence of laser irradiation (808 nm, 1.0 W cm^−2^, 5 min). As shown in Figure 3(E), less than 9% of DOX was released from the BSA-IrO_2_@DOX at pH 7.4 after 24 h, while the released DOX amount reached ∼28% at pH 5.0 after 24 h, which can be attributed to the reduction of the electrostatic interaction between DOX molecules and BSA. Moreover, the effect of NIR laser irradiation on the DOX release was further examined. It can be found that DOX is quickly released and the cumulative release drastically increased to ≈46% and ≈68% at pH 7.4 and 5.0 upon laser irradiation, respectively. The NIR-triggered DOX release from BSA-IrO_2_@DOX is mainly ascribed to the local temperature induced by BSA-IrO_2_ NPs under NIR laser irradiation could disintegrate the DOX molecules from the nanoplatform. Such pH- and NIR-responsive drug release capacity could avoid premature release during the circulation, which presented a potential approach for on demand drug delivery by this albumin-biomineralized nanocomposite.

### *In vitro* cellular uptake of BSA-IrO_2_@DOX NPs

**Figure 4.**
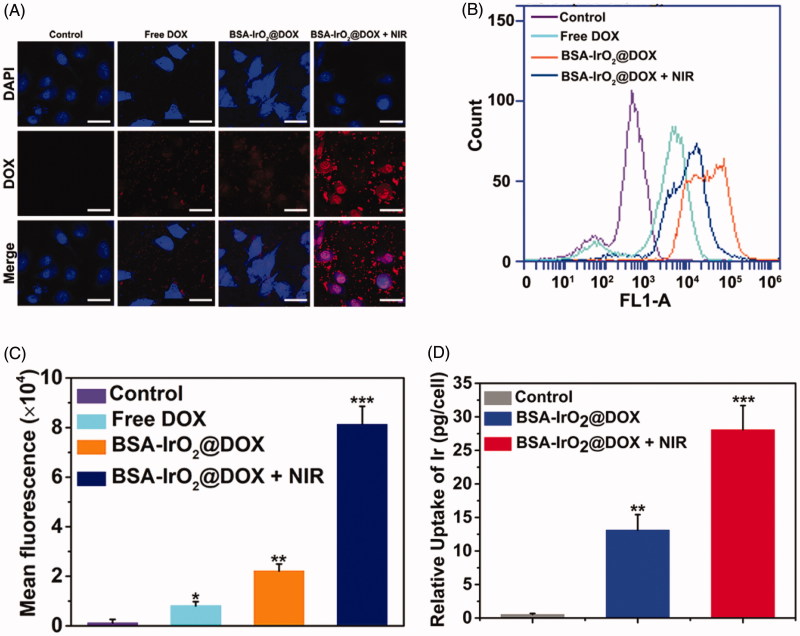
*In vitro* cellular uptake of BSA-IrO_2_@DOX NPs. (A) Confocal images of Saos-2 cells incubated with DMEM medium, free DOX, and BSA-IrO_2_@DOX with or without NIR irradiation (1.0 W cm^−2^) (relative DOX concentration: 5 μg/mL). Scale bar: 50 μm. (B) Flow cytometry data obtained on of Saos-2 cells incubated with PBS, DOX, and BSA-IrO_2_@DOX with or without NIR irradiation (1.0 W cm^−2^) and (C) the quantitative mean fluorescence intensities. (D) The uptake of Ir a by Saos-2 cells after treatment with the BSA-IrO_2_@DOX with or without NIR irradiation (1.0 W cm^−2^).

The localization of nanoparticles was investigated by CLSM (Figure 4(A)). Considerable fluorescent signal was detected in BSA-IrO_2_@DOX, whereas free DOX exhibited significant weak fluorescence. The weaker fluorescent signal of free DOX was clearly ascribed to the lower cellular uptake. In comparison, the fluorescent signal was further enhanced after adding laser irradiation, because heat induced by IrO_2_ upon NIR laser irradiation can enhance the cell uptake of nanoparticles. The intracellular drug release was studied by incubating Saos-2 cells with the BSA-IrO_2_@DOX NPs, followed by flow cytometry analysis. As shown in Figure 4(B,C), weak red fluorescence signal from the DOX molecules was observed for the cells incubated with free DOX. By contrast, the fluorescence intensities of cells treated with BSA-IrO_2_@DOX were much stronger than that free DOX group, confirmed that BSA-IrO_2_@DOX could be efficiently uptake by Saos-2 cells. Similiarly, the DOX signal was enhanced after adding laser irradiation, which could be attributed to the heat induced by laser can enhance the cell uptake of NPs, as well as greatly promoted the DOX release (Lu et al., 2017). To further evaluate the cellular uptake ability of the NPs, the Ir uptake within the Saos-2 cells after different treatments were quantitatively analyzed by ICP-AES. The uptake of Ir in NIR group was nearly 50-fold higher than that of the control group, and 2.5-fold of that in BSA-IrO_2_@DOX in the absence of NIR laser irradiation (Figure 4(D)). Therefore, the fabricated nanoplatform could be internalized in cancer cells and laser irradiation can further enhance the endocytosis amount.

### *In vitro* chemo-PTT synergistic effect of BSA-IrO_2_@DOX

**Figure 5.**
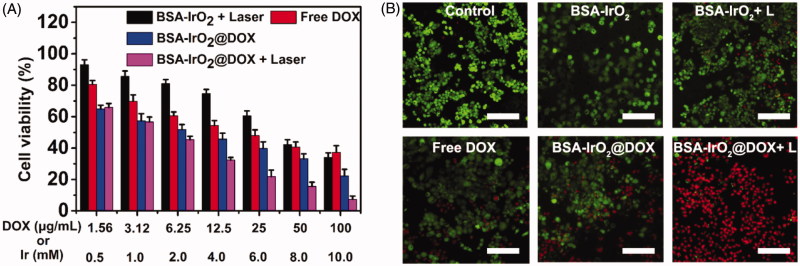
*In vitro* cell experiments. (A) Relative viabilities of Saos-2 cells after treatment with various groups. (B) Corresponding images of Saos-2 cells stained with calcein AM (live cells, green fluorescence) and PI (dead cells, red fluorescence). Scale bars = 200 µm.

Encouraged by the above results, one can imagine that the enhanced uptake of BSA-IrO_2_@DOX NPs would significantly enhance the chemotherapeutic efficacy against tumor cells. Biocompatibility of nanoparticles is a primary condition for biomedical applications. The *in vitro* cytotoxicity of BSA-IrO_2_ was examined in Saos-2 and A549 cells and a standard CCK-8 assay was performed to investigate the potential cytotoxicity of BSA-IrO_2_ NPs. As shown in Figure S5, no obvious toxicity of BSA-IrO_2_ in these two cell lines was observed, even at high Ir concentrations of 10 mM after incubation for 24 h, indicating the good biocompatibility of BSA-IrO_2_ NPs. Subsequently, the synergistic chemo-PTT therapeutic performance of BSA-IrO_2_@DOX was further evaluated. Saos-2 cells were treated with free DOX and BSA-IrO_2_@DOX at increasing concentrations for cell viability test. A DOX concentration-dependent cytotoxicity was observed in the BSA-IrO_2_@DOX and free DOX group. Also, the cell viabilities in the BSA-IrO_2_@DOX group were lower than that in free DOX groups. More importantly, The half maximal inhibitory concentration (IC_50_) of BSA-IrO_2_@DOX is 6.21 µg mL^−1^, while the free DOX was detected at 19.24 µg mL^−1^, indicating remarkable antitumor effect of BSA-IrO_2_@DOX. These results were mainly due to the low uptake of free DOX as proved in flow cytometry analysis (Figure 5(A)). Notably, over 90% of the cells died at a DOX concentration of 100 µg mL^−1^ when the cells treated with BSA-IrO_2_@DOX under NIR irradiation (1.0 W cm^−2^, 5 min), confirming the high efficacy of synergistic chemo-photothermal therapy strategy. The chemo-photothermal antitumor efficiency of BSA-IrO_2_@DOX NPs was further evaluated by calcein AM/PI assay. Almost all cells remained alive were observed in control and BSA-IrO_2_ groups (Figure 5(B)). When the two treatments were combined, most of the cells were dead, which is consistent with the CCK-8 results.

### *In vivo* therapeutic efficacy

**Figure 6.**
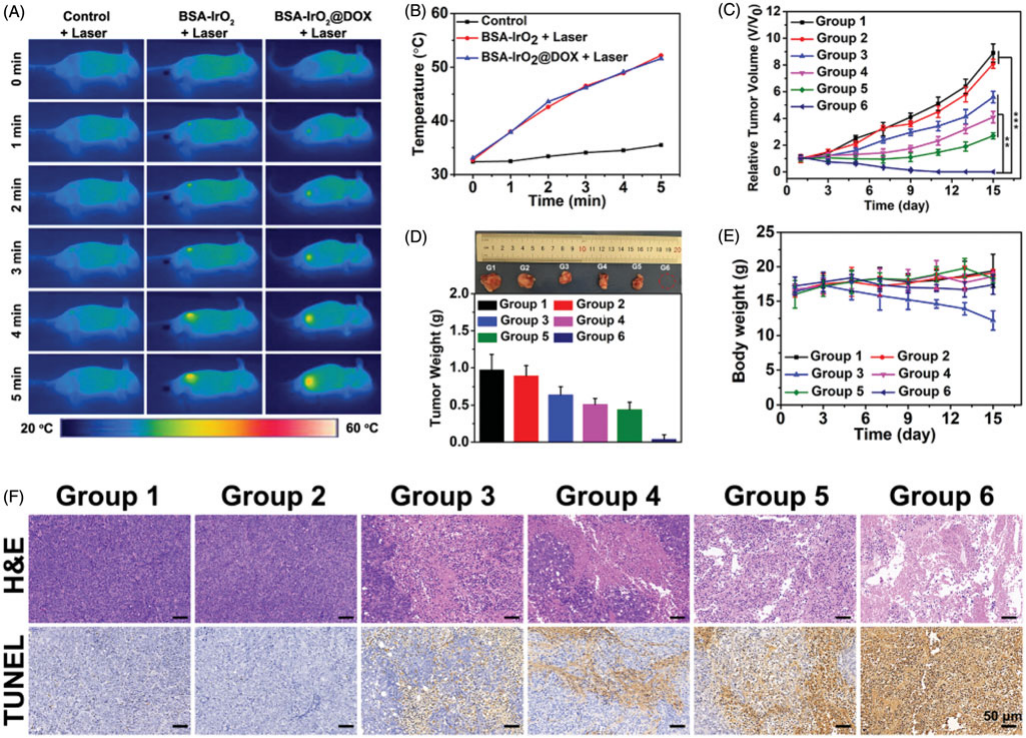
*In vivo* thermal imaging and combined cancer therapy effect of BSA-IrO_2_@DOX NPs. (A) Infrared thermographic maps and (B) time-dependent temperature changes in the Saos-2 tumor-bearing nude mice after treatment with control (PBS + laser), BSA-IrO_2_+laser, and BSA-IrO_2_@DOX + laser. (C) Tumor growth curves of Saos-2 tumor bearing nude mice after different treatments (**p < .01, and ***p < .001). (D) Tumor weights of mice at the 15th day after the treatments and digital photographs of corresponding excised tumors after different treated groups. (E) Time-dependent body-weight curves. (F) H&E-stained and TUNEL-stained of tumor tissues from Saos-2 tumor bearing mice after different treatments. Group 1: PBS; Group 2: BSA-IrO_2_ NPs; Group 3: Free DOX; Group 4: BSA-IrO_2_+laser; Group 5: BSA-IrO_2_@DOX; Group 6: BSA-IrO_2_@DOX NPs + laser.

Illuminated by the *in vitro* appreciable results, the possibilities of using the BSA-IrO_2_@DOX NPs as a synergistic therapeutic nanoplatform for *in vivo* application was further explored. To do this, we first established the Saos-2 xenograft tumor model and the mice were randomized into 6 groups: control, BSA-IrO_2_ NPs, free DOX, BSA-IrO_2_@DOX, BSA-IrO_2_ + laser and BSA-IrO_2_@DOX + laser. For the laser treated groups, the mice were irradiated with NIR laser (1 W cm^−2^, 5 min) after 12 h intravenous administration of BSA-IrO_2_ or BSA-IrO_2_@DOX dispersion. The temperature changes of the tumor sites in different groups were monitored using an infrared thermal camera (Figure 6(A)). Figure 6(B) shows that the tumor temperature in both BSA-IrO_2_ and BSA-IrO_2_@DOX groups could obviously increase to exceed 50 °C upon laser irradiation, whereas a tiny temperature change increased by <4 °C was observed in the PBS group after NIR irradiation. This indicates the BSA-IrO_2_ NPs could effectively convert the 808 nm laser into heat for thermal ablation of solid tumors in vivo.

To quantitatively estimate the antitumor efficacy, the tumor size was measured every 3 days during the following feeding period of two weeks. As shown in Figure 6(C), negligible inhibition effect on tumor growth was observed in both PBS and BSA-IrO_2_ group. As expected, clear inhibitory effect on tumor growth was obtained after treated with free DOX, BSA-IrO_2_@DOX and BSA-IrO_2_ plus laser irradiation. Also, it should be noted that better therapeutic effects were seen in BSA-IrO_2_@DOX and BSA-IrO_2_ + laser group than in free DOX group, suggesting the potential application of our BSA-IrO_2_ as drug delivery carrier and photothermal conversion agent. Notably, extremely high inhibition of tumor growth was shown in mice receiving BSA-IrO_2_@DOX NPs + laser treatment, which clearly indicated the synergistic effect of chemotherapy and photothermal therapy. The desirable synergistic chemo-PTT performance was also confirmed by representative tumor images and the tumor weight at the end of treatments (Figure 6(D)). Meanwhile, the body weight in free DOX group decreased by 25.1%, indicating the potential systemic toxicity of free DOX. However, no obvious weight loss were noted during other five treatments (Figure 6(E)), demonstrating the good biocompatibility of BSA-IrO_2_@DOX NPs. Afterwards, histological analysis (H&E and TUNEL staining) was conducted to further reveal the mechanism of the therapeutic efficacy (Figure 6(F)). For H&E staining analysis, the purple areas (nucleus stained) in BSA-IrO_2_@DOX NPs + laser group was much less than other groups, indicating the most severe damage of Saos-2 cells after synergistic chemo-PTT. Similar with H&E staining results, TUNEL images revealed the largest apoptotic cells presented (brown-staining cells) in group that treated with BSA-IrO_2_@DOX NPs + laser irradiation. These results clearly showed that, although the PTT- or chemotherapy-alone treatment identified favorable inhibition effect, it was much less effective than the synergistic therapy. Furthermore, the major organs including heart, spleen, liver, kidney and lung of the mice after treatment were collected and stained with H&E to investigate the in vivo toxicity (Figure S6). Clearly, no noticeable signal of inflammation or tissue damage was occurred in major organs from each group that treated with BSA-IrO_2_@DOX or BSA-IrO_2_, indicating that this multifunctional nanoplatform have excellent biosafety *in vivo*.

### *In vivo* blood circulation and biodistribution

Encouraged by the ideal *in vivo* anticancer performance of BSA-IrO_2_@DOX, the blood circulation time and biodistribution were further evaluated. The plasma levels of Ir were determined by ICP-AES at different time intervals following tail vein injection of BSA-IrO_2_@DOX NPs (20 mg kg^−1^). The blood levels of Ir concentrations decreased gradually over time and the blood circulation half time of BSA-IrO_2_@DOX was calculated to be 4.24 h (Figure S7). The prolonged circulation time of BSA-IrO_2_@DOX can facilitate their subsequent tumor accumulation *via* the EPR effect. For *in vivo* biodistribution study, BSA-IrO_2_@DOX NPs were administered to the Saos-2 tumor-bearing mice via intravenous injection at a dose of 20 mg kg^−1^, followed by measurements of Ir in tumor and different tissues at varied time intervals. It can be observed that BSA-IrO_2_@DOX could efficiently accumulate in tumor tissues and reached 9.5% ID/g at 24 h (Figure S8) after intravenous injection as determined by ICP-AES test, which can be ascribed to the tumor EPR effect. Meanwhile, high levels of BSA-IrO_2_@DOX NPs accumulated in liver and spleen was found, which could be attributed to the absorption of the reticuloendothelial system (Yang et al., 2016). Obviously, BSA-IrO_2_@DOX exhibit efficient tumor accumulation due to their prolonged circulation time and passive targeting effect.

## Conclusion

In conclusion, we have successfully developed a biocompatible nanoplatform based on BSA-IrO_2_ NPs for stimuli-responsive drug delivery and synergistic chemo-photothermal therapy. The BSA-IrO_2_ NPs was prepared through a simple biomineralization method. The as-prepared BSA-IrO_2_ NPs exhibit superb biocompatibility and excellent photothermal conversion capability upon NIR irradiation. The NPs can also be served as carrier for loading of small molecular drugs. Using DOX as a model drug, the BSA-IrO_2_ also showed considerable drug loading capacity, and the drug release could be triggered by NIR laser and acidic pH. Importantly, highly effective synergetic antitumor efficacy of the BSA-IrO_2_@DOX has been demonstrated both *in vitro* and *in vivo*, which is superior to that of either monotherapy alone. Further, *in vivo* experiments showed that BSA-IrO_2_@DOX had long blood circulation time and efficient tumor accumulation. Thus, such BSA-IrO_2_@DOX nanoparticles present great potential as a nanoplatform for the development of more efficient antitumor treatments. This work has focused on the direction of iridium-based nanomaterials for cancer therapy. Considering that this is the first time to employ the BSA-IrO_2_ nanoparticles as drug carrier, the protein-based nanocarriers strategy in this work shows general potential for the fabrication of other drug delivery systems.
